# Acute Hemoperitoneum after Administration of Prostaglandin E_2_ for Induction of Labour

**DOI:** 10.1155/2015/659274

**Published:** 2015-10-01

**Authors:** Zhenyu Zhang, Jiangyan Lou

**Affiliations:** Department of Gynecology and Obstetrics, West China Second Hospital, Sichuan University, Chengdu, Sichuan 610041, China

## Abstract

Prostaglandin E_2_ is widely used in obstetrics and is thought to be relatively safe for cervical ripening and induction of labour. Here we present a case in which acute hemoperitoneum was observed after administration of prostaglandin E_2_ in a pregnant woman. The patient had a history of endometriosis, and a severe pelvic adhesion (ASRM stage IV) was found during her last laparoscopic surgery 3 years previously. In cases with endometriosis, use of prostaglandin E_2_ for induction of labour in pregnant women must be done cautiously.

## 1. Introduction

Endometriosis is usually a common gynaecologic condition. It is associated with chronic inflammation, which causes tissue injury, painful symptoms, and infertility [1]. In recent years, prostaglandin E_2_ (PGE_2_) is widely used for induction of labour around the world [2]. However, data on the safety of PGE_2_ in pregnant women with endometriosis is deficient. The case of acute hemoperitoneum after intravaginal administration of PGE_2_ for cervical ripening has been rarely reported. Here we can learn that induction of labour with PGE_2_ in women with endometriosis may be associated with acute hemoperitoneum.

## 2. Presentation of the Case

**Figure 1:**
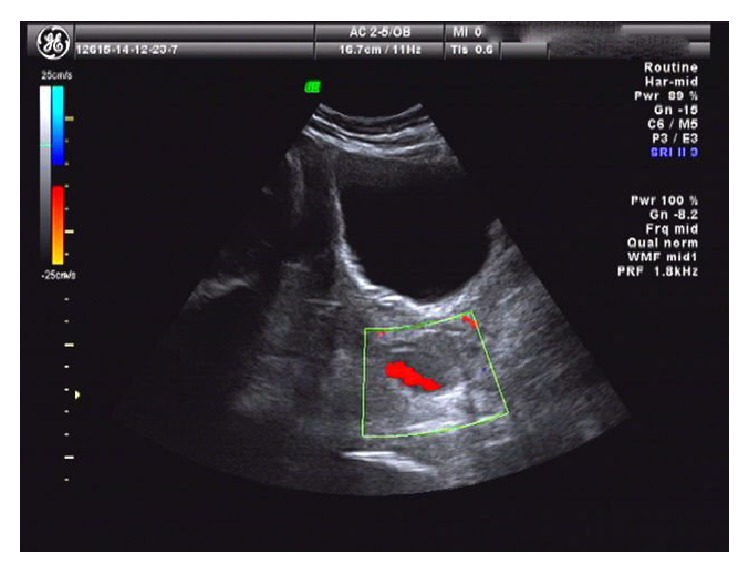
Sonographic findings showing unilocular cysts containing hyperechoic fluid and irregular wall with some blood flow signals.

A 25-year-old woman (G1P0) was admitted to the hospital at 41 weeks of gestation with a 5-year history of endometriosis. Laparoscopic excision of endometriotic lesions had been performed in this hospital 3 years previously. Ultrasound demonstrated that there was an ovarian endometrioma with a diameter of 3.8 cm on her right ovary (Figure 1). The endometrioma had been found in antenatal examination without enlargement during pregnancy.

She felt no contractions and denied having vaginal bleeding. Clinical pelvimetry and foetal size and presentation were examined before initiating attempts to induce labour. Her Bishop score was 4, so PGE_2_ preparations were inserted into the posterior fornix at 9:20 a.m. The patient had symptoms of regular abdominal pain from 11:30 a.m. And then, the cardiotocography recorded six contractions lasting 40 to 50 seconds per time within 10 minutes around 12 p.m. Her blood pressure was 95/58 mmHg and her pulse rate was 100 beats per min. However, it was not helpful to reverse the effects of hyperstimulation and tachysystole in the hour following removal of the PGE_2_ vaginal insert. Vaginal examinations subsequently identified that the cervix was still closed. Meanwhile, she complained of persistent inferior abdominal pain with symptoms including nausea and vomiting. Her blood pressure rose to 120/65 mmHg, and her pulse rate increased to 110 beats per min.

An emergency caesarean delivery was scheduled at 3 p.m. When the peritoneal cavity was entered, a hemoperitoneum was clearly seen, which consisted of fresh liquid and clotted blood scattered into the intestinal walls and mesentery. About 1000 mL of blood and 1500 g of clotted blood were evacuated. The uterine wall was then transversely incised, the foetus was extracted, and the placenta was routinely separated from the uterus. The placenta appeared normal and complete. We excluded the possibility of placental abruption after further examination. The new born baby survived, with an Apgar score of  7 and 10 at the first and fifth min. Then adhesions were extensively detected between the bilateral adnexa, omentum majus, and uterus. Part of the ruptured cyst and the right ovary were tightly adhered to the posterior uterine wall. The region of the adhesion on the surface of the uterus appeared as an inflammatory exudation with small bleeding vessels. Active bleeding from the ovarian cyst wall and posterior uterine wall vessels was observed. The maternal haemoglobin levels dropped significantly to 6.3 g/dL from the initial 9.2 g/dL.

**Figure 2:**
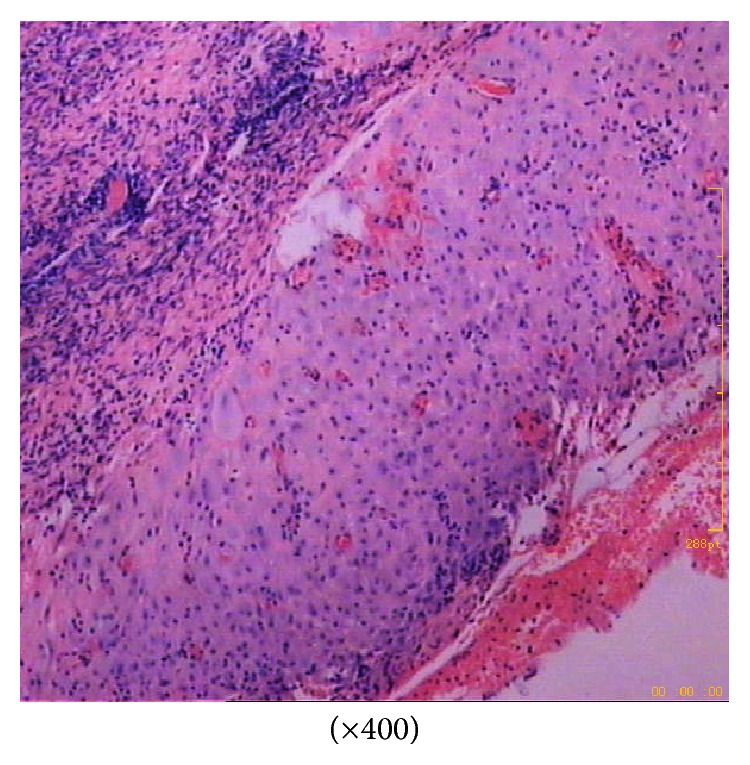
Histopathological view showing endometrial glands and stroma with extensive decidualization.

After adhesiolysis and ovarian cystectomy, the original region that was bleeding was sutured, and then haemostasis was achieved. Haemorrhage from the left ovary and the upper abdomen was excluded through a more comprehensive pelvic and abdominal exploration. The patient received a blood transfusion consisting of six units of packed red blood cells and 800 mL fresh frozen plasma, which temporarily corrected her anaemic condition. The postoperative histological examination from the ovarian cyst proved to be similar to the endometrium with signs of decidualization (Figure 2). She was discharged on the seventh postoperative day without any complications.

## 3. Discussion 

Our findings support those of the previous reports that endometriosis might be related to acute spontaneous hemoperitoneum. Janicki et al. reported a case of hemoperitoneum secondary to the rupture of an ovarian endometrioma in a nonpregnant woman with severe endometriosis [3]. In contrast, there was an abundant blood supply in the enlarged uterus in the present case during a gestation of 41 weeks. Dilation of the vessels and accelerated blood flow increased the risk of vascular rupture. Also, a hemoperitoneum caused by a ruptured ovarian endometrioma was reported in 2011. In that case, the woman was pregnant with twins and had a laparotomy at 27 weeks of gestation [4].

Aziz et al. tried to explain the aetiology of acute hemoperitoneum [5]. One possible reason given was that venous pressure is increased in the utero-ovarian circulation during muscular activity and straining during labour. Inoue et al. suggested two possible explanations for the aetiology. One was chronic inflammation and the other was the resultant adhesion [6]. The patient in our present case had a 5-year history of endometriosis, and the last laparoscopic surgery has not relieved pelvic adhesions. As inflammatory mediators were released and the pelvic anatomical structure changed, intense uterine contractions forced the vessels in the adhesion layer to rupture.

The size of the ovarian endometrioma is another question of concern. The potential for vessels to rupture becomes more serious as the cyst grows larger. To the best of our knowledge, the 3.8 cm ovarian cyst in our case is the smallest reported to have ruptured in a pregnant woman. We conclude that the severity of the pelvic adhesion and the intensity of uterine contractions are more important than the size of the ovarian cyst.

Evidence showed that one of the most common adverse effects of PGE_2_ was uterine tachysystole [1]. Cases of hyperstimulation and subsequent uterine rupture following its application have been reported [7], but the prostaglandin-induced rupture of ovarian and uterine vessels is rare. According to clinical management guidelines for the induction of labour in Canada [1] or the United States [8], PGE_2_ is not contraindicated in patients with ovarian endometrioma. During general labour without PGE_2_, uterine contractions also tend to pull the uterus away from the ovaries and increase the blood pressure in the utero-ovarian circulation. In our case, more than 5 contractions triggered by PGE_2_ over a 10-minute period obviously increased the risk of vessel rupture.

This case illustrates several points that may prove useful to clinicians when dealing with these conditions. Induction of labour with PGE_2_ may be associated with acute hemoperitoneum. Therefore, we should be very cautious when PGE_2_ is used to induce labour in women with endometriosis, particularly when the patient has a history of severe pelvic adhesions.
